# Unleashing the potential of high-throughput sequencing for plant virus and viroid detection in Mexico

**DOI:** 10.3389/fmicb.2025.1603010

**Published:** 2025-05-15

**Authors:** Carolina Pacheco-Dorantes, Juan Manuel Tovar-Pedraza, Daniel Leobardo Ochoa-Martínez, Ramiro González-Garza, Alfredo Diaz-Lara

**Affiliations:** ^1^School of Engineering and Sciences, Tecnologico de Monterrey, Santiago de Querétaro, Querétaro, Mexico; ^2^Laboratorio de Fitopatología, Coordinación Regional Culiacán, Centro de Investigación en Alimentación y Desarrollo, Culiacán, Sinaloa, Mexico; ^3^Posgrado en Fitosanidad – Fitopatología, Colegio de Postgraduados, Texcoco, Estado de México, Mexico; ^4^BioCiencia S.A. de C.V., Monterrey, Nuevo León, Mexico

**Keywords:** high-throughput sequencing, plant virology, metagenomics, plant disease, diagnostics, viroid, virus

## Abstract

High-throughput sequencing (HTS) has revolutionized plant virology in Mexico by enhancing the detection and characterization of plant viruses and viroids. This technology has contributed to identifying previously neglected pathogens affecting key crops such as corn, beans, and tomato. The use of HTS has also revealed the presence of mixed viral infections, highlighting the complexity of plant viromes within agricultural ecosystems. Furthermore, metagenomic studies have demonstrated the role of water sources as reservoirs for plant viruses, underscoring the urgent need for improved management strategies. Despite its advantages, the widespread adoption of HTS faces challenges, including high costs, the need for bioinformatics expertise, and infrastructure limitations. Supporting collaborations between research institutions and regulatory agencies is crucial to integrating HTS into routine phytosanitary programs. Future research should aim to expand HTS applications to include epidemiological monitoring, resistance breeding, and the development of sustainable management strategies to mitigate the impact of emerging plant viruses in Mexico.

## 1 Introduction

The history of plant virology in Mexico is closely linked to the country’s agricultural development and the ongoing challenges posed by plant diseases. In the mid-20th century, Mexican scientists began to identify and describe viral pathogens affecting important crops such as corn/maize (*Zea mays*), common bean (*Phaseolus vulgaris*), and tomato (*Solanum lycopersicum*).^1^ These crops are not only staples in the Mexican diet but also key products for the national economy. These early efforts laid the foundation for a solid field of study, further strengthened by the establishment of several research institutions along the country and the development of diagnostic tools and management strategies by the National Service for Health, Safety and Food Quality (Servicio Nacional de Sanidad, Inocuidad y Calidad Agroalimentaria, SENASICA).

Over the last seven decades, the field of plant virology in Mexico has grown in both scope and complexity. Adopting serological and molecular techniques during the late 20th and early 21st centuries allowed for more precise identification and characterization of plant viruses (Massart et al., 2014; Rithesh et al., 2025). These tools have proven to be essential for tracking virus dynamics in plant populations, providing information on transmission patterns and geographic dispersion (Rodríguez-Negrete et al., 2019; Claverie et al., 2017). Thus, Mexican scientists have played a significant role in these developments, often collaborating with international partners to enhance the country’s capacity to manage plant viral diseases (González-Pérez et al., 2024; Alcalá-Briseño et al., 2020; Maliogka et al., 2018). Today, plant virology stands as an integral part of agricultural research in Mexico, with ongoing efforts focused on developing resistant crop varieties and protecting the country’s agricultural heritage from a continually evolving array of viral pathogens (Ortega-Piña et al., 2024; Martínez-Marrero et al., 2020).

The accurate diagnosis of plant viruses is crucial for minimizing their impact on agriculture. Viral infections can significantly reduce crop yields and quality, leading to substantial economic losses (Nizamani et al., 2023). In a diverse agricultural country like Mexico, timely identification of plant pathogens is essential not only to safeguard staple crops but also to protect economically valuable plants, such as avocado (*Persea americana*), agave (*Agave* spp.), and strawberry (*Fragaria* × *ananassa*; Diaz-Lara et al., 2021; Martínez-Marrero et al., 2020). Traditional diagnostic techniques, such as bio-indexing, enzyme-linked immunosorbent assay (ELISA) and polymerase chain reaction (PCR), have long been used in plant virology. However, these methods are limited to detecting known pathogens, for which indicator plants, antibodies and specific primers are available, leaving emerging or unidentified infections undetected (Mehetre et al., 2021).

**Figure 1:**
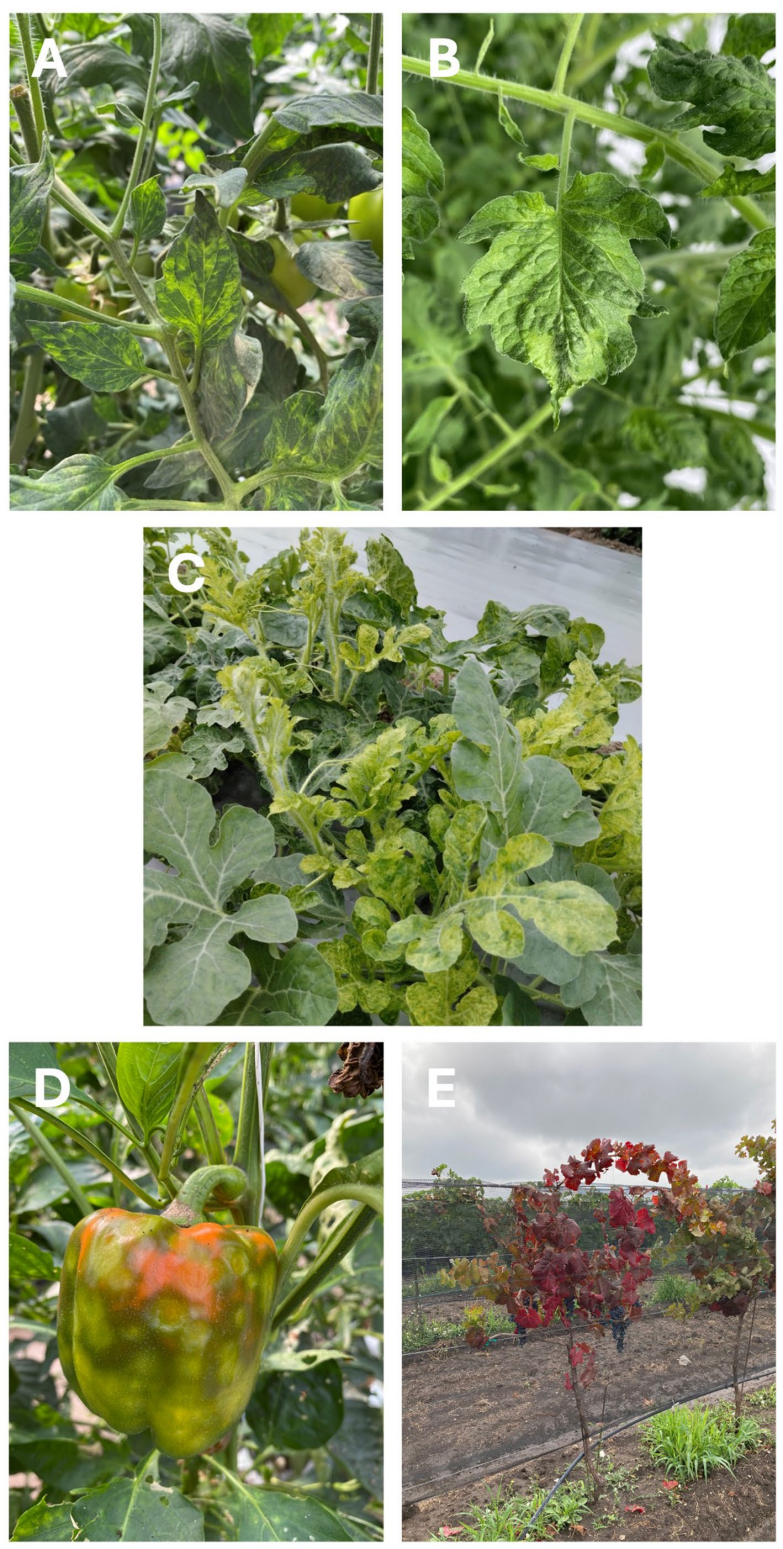
Multiple viruses have been identified in symptomatic plants by high-throughput sequencing. **(A)** Tomato with characteristic symptoms of pepino mosaic virus (PepMV) infection. **(B)** Tomato leaf with symptoms of tomato brown rugose fruit virus (ToBRFV). **(C)** Watermelon with a mixed infection by cucurbit yellow stunting disorder virus (CYSDV) and watermelon chlorotic stunt virus (WmCSV). **(D)** Bell pepper showing symptoms of tomato spotted wilt virus (TSWV). **(E)** Grapevine infected by grapevine leafroll-associated virus 3 (GLRaV-3).

High-throughput sequencing (HTS) has revolutionized the field of plant virology by providing unprecedented sensitivity and accuracy in detecting a wide variety of viruses and viroids (Figure 1; Saeed et al., 2023; Raza and Shahid, 2020). Unlike traditional methods such as ELISA and PCR, HTS enables the identification of both known and novel viruses without prior knowledge of the target (Rivarez et al., 2023; Kutnjak et al., 2021). Its capacity to simultaneously analyze multiple infections and provide a comprehensive overview of a plant’s health makes it an invaluable tool for studying complex and emerging viral threats (Kanapiya et al., 2024; Villamor et al., 2019; Diaz-Lara et al., 2023). For example, a study conducted in the Czech Republic demonstrated the power of HTS by analyzing grapevine (*Vitis* spp.) samples (Eichmeier et al., 2016). This research revealed the presence of viruses and viroids previously unreported in the country, including grapevine rupestris vein feathering virus (GRVFV) and grapevine yellow speckle viroid 1 (GYSVd1, *Apscaviroid alphaflavivitis*), highlighting the potential of this technology to discover hidden pathogens. Furthermore, HTS has significantly expanded our understanding of viral diversity present in agricultural ecosystems, revealing interactions between viruses, their hosts, and vectors. This approach allows for decoding virome structures in specific crops and locations, which is critical for identifying ecological factors that favor the emergence or re-emergence of viral epidemics (Lopez-Roblero et al., 2023; Ortega-Acosta et al., 2023a).

Despite its advantages, HTS still has challenges. The technique generates large volumes of data, requiring advanced bioinformatics expertise for analysis, which can represent a barrier for some laboratories (Villamor et al., 2019; Mutz et al., 2013). Additionally, while the cost of HTS per sample has decreased over time, the initial investment in sequencing platforms and data processing infrastructure remains relatively high, making it less accessible for routine diagnostics (Kanapiya et al., 2024; Rithesh et al., 2025). Nonetheless, the comprehensive detection capabilities of HTS make it a valuable complement to traditional methods, particularly in virus-viroid discovery and characterization (Ortega-Acosta et al., 2024a,b).

The integration of HTS into plant virology has revolutionized the understanding and management of viral diseases in economically significant crops worldwide. Its application across various agricultural systems has revealed the complexity of plant viromes and provided valuable insights that can inform the development of more effective disease management strategies (Salgado-Ortíz et al., 2020; Rodríguez-Verástegui et al., 2022). These advancements emphasize the necessity of incorporating HTS into Mexico’s phytosanitary frameworks, particularly in the face of global trade and climate change challenges.

## 2 High-throughput sequencing of plant viruses in Mexico

The application of sequencing technologies has significantly advanced the discovery of plant viruses in Mexico, a country with diverse agricultural landscapes and critical crops prone to viral threats. Thus, research efforts in Mexico have highlighted the power of HTS to unveil the hidden virome in agricultural and natural systems, contributing to a deeper understanding of plant viral diversity and ecology. This section explores identified viruses and viroids in Mexico via HTS (Table 1), shedding light on their discovery through cutting-edge molecular techniques and their potential implications for agricultural research and management.

**Table 1:** Plant viruses and viroids identified in Mexico via high-throughput sequencing (HTS).

Name of viral agent	Acronym	Species name	Genus	Host	Reference
Alfalfa mosaic virus	AMV	*Alfamovirus AMV*	*Alfamovirus*	Alfalfa (*Medicago sativa*)	Lopez-Roblero et al. (2023)
Bean calico mosaic virus	BCaMV	*Begomovirus phaseolicaliconis*	*Begomovirus*	*Brassicaceae*	Rodríguez-Negrete et al. (2019)
Bean common mosaic necrosis virus	BCMNV	*Potyvirus phaseoli*	*Potyvirus*	Wild and domesticated beans (*Phaseolus* spp.)	Chiquito-Almanza et al. (2021)
Bean common mosaic virus	BCMV	*Potyvirus phaseovulgaris*	*Potyvirus*	Wild and domesticated beans (*Phaseolus* spp.)	Chiquito-Almanza et al. (2021)
Bean golden yellow mosaic virus	BGYMV	*Begomovirus birdi*	*Begomovirus*	Wild and domesticated beans (*Phaseolus* spp.)	Chiquito-Almanza et al. (2021)
Bean yellow mosaic Mexico virus	BYMMV	*Begomovirus phaseolimexicoense*	*Begomovirus*	*Fabaceae*	Rodríguez-Negrete et al. (2019)
Beet curly top virus	BCTV	*Curtovirus betae*	*Curtovirus*	*Amaranthaceae*	Rodríguez-Negrete et al. (2019)
Blechum interveinal chlorosis virus	BleICV	*Begomovirus blechi*	*Begomovirus*	*Acanthaceae*	Rodríguez-Negrete et al. (2019)
Brome mosaic virus	BMV	*Bromovirus BMV*	*Bromovirus*	Maize (*Zea mays*)	Lappe et al. (2022)
Cabbage leaf curl virus	CabLCV	*Begomovirus brassicae*	*Begomovirus*	*Brassicaceae*	Rodríguez-Negrete et al. (2019)
Chilli leaf curl virus	ChiLCV	*Begomovirus chillicapsici*	*Begomovirus*	*Solanaceae*	Rodríguez-Negrete et al. (2019)
Cowpea mild mottle virus	CPMMV	*Carlavirus vignae*	*Carlavirus*	Wild and domesticated beans (*Phaseolus* spp.)	Chiquito-Almanza et al. (2021)
Cucumber green mottle mosaic virus	CGMMV	*Tobamovirus viridimaculae*	*Tobamovirus*	Cucumber (*Cucumis sativus*)	Lopez-Roblero et al. (2023)
Cucurbit yellow stunting disorder virus	CYSDV	*Crinivirus cucurbitae*	*Crinivirus*	Watermelon (*Citrullus lanatus)*	Hernández-Pérez et al. (2025)
Euphorbia mosaic virus	EuMV	*Begomovirus euphorbiamusivi*	*Begomovirus*	*Euphorbiaceae*	Rodríguez-Negrete et al. (2019)
Euphorbia yellow mosaic virus	EuYMV	*Begomovirus euphorbiamusiviflavi*	*Begomovirus*	*Euphorbiaceae*	Rodríguez-Negrete et al. (2019)
Galium leaf distortion virus	GLDV	*Begomovirus galii*	*Begomovirus*	*Galium* sp.	Guevara-Rivera et al. (2022)
Grapevine asteroid mosaic-associated virus	GAMaV	*Marafivirus asteroides*	*Marafivirus*	Grapevine (*Vitis vinifera*)	Diaz-Lara et al. (2023)
Grapevine Cabernet Sauvignon reovirus	GCSV	Without binomial name	*Reovirus*	Grapevine (*Vitis vinifera*)	Diaz-Lara et al. (2023)
Grapevine enamovirus 2	GEV2	Without binomial name	*Enamovirus*	Grapevine (*Vitis vinifera*)	Diaz-Lara et al. (2023)
Grapevine fanleaf virus	GFLV	*Nepovirus foliumflabelli*	*Nepovirus*	Grapevine (*Vitis vinifera*)	Diaz-Lara et al. (2023)
Grapevine fleck virus	GFkV	*Maculavirus vitis*	*Maculavirus*	Grapevine (*Vitis vinifera*)	Diaz-Lara et al. (2023)
Grapevine hammerhead viroid-like RNA	GHVd	Without binomial name	*Avsunviroid*	Grapevine (*Vitis vinifera*)	Diaz-Lara et al. (2023)
Grapevine leafroll-associated virus 1	GLRaV-1	*Ampelovirus univitis*	*Ampelovirus*	Grapevine (*Vitis vinifera*)	Diaz-Lara et al. (2023)
Grapevine leafroll-associated virus 2	GLRaV-2	*Closterovirus vitis*	*Closterovirus*	Grapevine (*Vitis vinifera*)	Diaz-Lara et al. (2023)
Grapevine leafroll-associated virus 3	GLRaV-3	*Ampelovirus trivitis*	*Ampelovirus*	Grapevine (*Vitis vinifera*)	Diaz-Lara et al. (2023)
Grapevine leafroll-associated virus 4	GLRaV-4	*Ampelovirus tetravitis*	*Ampelovirus*	Grapevine (*Vitis vinifera*)	Diaz-Lara et al. (2023)
Grapevine Pinot gris virus	GPGV	*Trichovirus pinovitis*	*Trichovirus*	Grapevine (*Vitis vinifera*)	Diaz-Lara et al. (2023)
Grapevine red globe virus	GRGV	Without binomial name	*Maculavirus*	Grapevine (*Vitis vinifera*)	Diaz-Lara et al. (2023)
Grapevine rupestris stem pitting-associated virus	GRSPaV	*Foveavirus rupestris*	*Foveavirus*	Grapevine (*Vitis vinifera*)	Diaz-Lara et al. (2023)
Grapevine rupestris vein feathering virus	GRVFV	Without binomial name	*Marafivirus*	Grapevine (*Vitis vinifera*)	Diaz-Lara et al. (2023)
Grapevine Syrah virus 1	GSyV-1	*Marafivirus syrahense*	*Marafivirus*	Grapevine (*Vitis vinifera*)	Diaz-Lara et al. (2023)
Grapevine virus B	GVB	*Vitivirus betavitis*	*Vitivirus*	Grapevine (*Vitis vinifera*)	Diaz-Lara et al. (2023)
Grapevine yellow speckle viroid 1	GYSVd1	*Apscaviroid alphaflavivitis*	*Apscaviroid*	Grapevine (*Vitis vinifera*)	Diaz-Lara et al. (2023)
Grapevine yellow speckle viroid 2	GYSVd2	*Apscaviroid betaflavivitis*	*Apscaviroid*	Grapevine (*Vitis vinifera*)	Diaz-Lara et al. (2023)
Grapevine yellow speckle viroid 3	GYSVd3	Without binomial name	*Apscaviroid*	Grapevine (*Vitis vinifera*)	Diaz-Lara et al. (2023)
Lettuce mosaic virus	LMV	*Potyvirus lactucae*	*Potyvirus*	Lettuce *(Lactuca sativa)*	Lopez-Roblero et al. (2023)
Maize chlorotic mottle virus	MCMV	*Machlomovirus zeae*	*Machlomovirus*	Maize (*Zea mays*)	Lappe et al. (2022)
Maize dwarf mosaic virus	MDMV	*Potyvirus zeananus*	*Potyvirus*	Maize (*Zea mays*)	Lopez-Roblero et al. (2023)
Maize rayado fino virus	MRFV	*Marafivirus maydis*	*Marafivirus*	Maize (*Zea mays*)	Lappe et al. (2022)
Maize yellow mosaic virus	MaYMV	*Polerovirus MAYMV*	*Polerovirus*	*Zea nicaraguensis*	Lappe et al. (2022)
Maize-associated tombusvirus	MaTV	Without binomial name	*Tombusviridae*	Maize (*Zea mays*)	Lappe et al. (2022)
Maize-associated totivirus 1	MATV1	*Totivirus shichi*	*Totivirus*	Maize (*Zea mays*)	Lappe et al. (2022)
Maize-associated umbra-like virus	MULV	Without binomial name	*Umbravirus*	Maize (*Zea mays*)	Lappe et al. (2022)
Malvastrum bright yellow mosaic virus	MaBYMV	*Begomovirus malvastrumflavi*	*Begomovirus*	*Malvaceae*	Rodríguez-Negrete et al. (2019)
Mexican Opuntia viroid	MOVd	Without binomial name	*Pospiviroid*	*Opuntia* spp.	Ortega-Acosta et al. (2024a,b)
North American maize-associated mastrevirus	NAMaMV	Without binomial name	*Mastrevirus*	Maize (*Zea mays*)	Lappe et al. (2022)
Okra yellow mosaic Mexico virus	OYMMV	*Begomovirus abelsmoschusmexicoense*	*Begomovirus*	*Malvaceae*	Rodríguez-Negrete et al. (2019)
Opuntia viroid 1	OVd-1	Without binomial name	*Apscaviroid*	*Opuntia* spp.	Ortega-Acosta et al. (2024a,b)
Opuntia viroid 2	OVd-2	Without binomial name	*Apscaviroid*	*Opuntia* spp.	Ortega-Acosta et al. (2024a,b)
Paprika mild mottle virus	PaMMV	*Tobamovirus paprikae*	*Tobamovirus*	Pepper (*Capsicum* spp.)	Lopez-Roblero et al. (2023)
Pepper golden mosaic virus	PepGMV	*Begomovirus capsicummusivi*	*Begomovirus*	*Solanaceae*	Rodríguez-Negrete et al. (2019)
Pepper huasteco yellow vein virus	PHYVV	*Begomovirus capsicumhuastecoense*	*Begomovirus*	Tomato (*Solanum lycopersicum*)	González-Pérez et al. (2024)
Pepper leafroll virus	PepLRV	*Begomovirus capsicumcontorsioris*	*Begomovirus*	*Solanaceae*	Rodríguez-Negrete et al. (2019)
Pepper mild mottle virus	PMMoV	*Tobamovirus capsici*	*Tobamovirus*	Pepper (*Capsicum* spp.)	Lopez-Roblero et al. (2023)
Phaseolus vulgaris alphaendornavirus 1	PvAEV-1	*Alphaendornavirus phaseoli*	*Alphaendornavirus*	Common bean (*Phaseolus vulgaris*)	Chiquito-Almanza et al. (2021)
Phaseolus vulgaris alphaendornavirus 2	PvAEV-2	*Alphaendornavirus fuphaseoli*	*Alphaendornavirus*	Common bean (*Phaseolus vulgaris*)	Chiquito-Almanza et al. (2021)
Potato yellow mosaic virus	PYMV	*Begomovirus tuberosi*	*Begomovirus*	*Solanaceae*	Rodríguez-Negrete et al. (2019)
Rattail cactus necrosis-associated virus	RCNaV	*Tobamovirus muricaudae*	*Tobamovirus*	*Cactaceae*	Lopez-Roblero et al. (2023)
Rhynchosia golden mosaic Sinaloa virus	RhGMSV	*Begomovirus rhynchosiasinaloaense*	*Begomovirus*	*Fabaceae*	Rodríguez-Negrete et al. (2019)
Rhynchosia golden mosaic virus	RhGMV	*Begomovirus rhynchosiaurei*	*Begomovirus*	*Fabaceae*	Rodríguez-Negrete et al. (2019)
Sida golden yellow vein virus	SiGYVV	*Begomovirus sidaureivenae*	*Begomovirus*	*Malvaceae*	Rodríguez-Negrete et al. (2019)
Sida mosaic Sinaloa virus	SiMSiV	*Begomovirus sidasinaloaense*	*Begomovirus*	*Solanaceae*	Rodríguez-Negrete et al. (2019)
Solanum mosaic Bolivia virus	SoMBoV	*Begomovirus solanumboliviense*	*Begomovirus*	*Solanaceae*	Rodríguez-Negrete et al. (2019)
Squash leaf curl virus	SLCV	*Begomovirus cucurbitapeponis*	*Begomovirus*	*Cucurbitaceae*	Rodríguez-Negrete et al. (2019)
Sweet potato leaf curl virus	SPLCV	*Begomovirus ipomoeae*	*Begomovirus*	*Convolvulaceae*	Rodríguez-Negrete et al. (2019)
Teosinte-associated betaflexivirus	TaBV	Without binomial name	*Betaflexivirus*	Teosinte (*Zea* spp.)	Lappe et al. (2022)
Tobacco etch virus	TEV	*Potyvirus nicotianainsculpentis*	*Potyvirus*	*Senna multiglandulosa*	Ortega-Acosta et al. (2023b)
Tobacco mild green mosaic virus	TMGMV	*Tobamovirus mititessellati*	*Tobamovirus*	*Solanaceae*	Lopez-Roblero et al. (2023)
Tobacco mosaic virus	TMV	*Tobamovirus tabaci*	*Tobamovirus*	Tobacco (*Nicotiana tabacum*)	Lopez-Roblero et al. (2023)
Tobacco ringspot virus	TRSV	*Nepovirus nicotianae*	*Nepovirus*	Blackberry (*Rubus* spp.)	Diaz-Lara et al. (2020)
Tomato brown rugose fruit virus	ToBRFV	*Tobamovirus fructirugosum*	*Tobamovirus*	Tomato (*Solanum lycopersicum*)	Lopez-Roblero et al. (2023)
Tomato chino La Paz virus	ToChLPV	*Begomovirus solanumlapazense*	*Begomovirus*	*Solanaceae*	Rodríguez-Negrete et al. (2019)
Tomato chlorosis virus	ToCV	*Crinivirus tomatichlorosis*	*Crinivirus*	Tomato (*Solanum lycopersicum*)	González-Pérez et al. (2024)
Tomato golden mosaic virus	ToGMoV	*Begomovirus solanumaureimusivi*	*Begomovirus*	Tomato (*Solanum lycopersicum*)	González-Pérez et al. (2024)
Tomato mosaic virus	ToMV	*Tobamovirus tomatotessellati*	*Tobamovirus*	Tomato (*Solanum lycopersicum*)	Lopez-Roblero et al. (2023)
Tomato pseudo-curly top virus	TPCTV	*Topocuvirus solani*	*Topocuvirus*	*Solanaceae*	Rodríguez-Negrete et al. (2019)
Tomato severe leaf curl virus	ToSLCV	*Begomovirus solanumseveri*	*Begomovirus*	*Solanaceae*	Rodríguez-Negrete et al. (2019)
Tomato spotted wilt virus	TSWV	*Orthotospovirus tomatomaculae*	*Orthotospovirus*	Tomato (*Solanum lycopersicum*)	González-Pérez et al. (2024)
Tomato yellow leaf curl virus	TYLCV	*Begomovirus coheni*	*Begomovirus*	Tomato (*Solanum lycopersicum*)	González-Pérez et al. (2024)
Tomato yellow spot virus	ToYSV	*Begomovirus solanumflavusmaculae*	*Begomovirus*	*Solanaceae*	Rodríguez-Negrete et al. (2019)
Tropical soda apple mosaic virus	TSAMV	*Tobamovirus tropici*	*Tobamovirus*	Tomato (*Solanum lycopersicum*)	Lopez-Roblero et al. (2023)
Tuberose mild mosaic virus	TuMMV	*Potyvirus polianthis*	*Potyvirus*	*Agave attenuata, Agave amica*	De la Torre-Almaraz et al. (2023)
Vigna yellow mosaic virus	ViYMV	*Begomovirus vignae*	*Begomovirus*	*Fabaceae*	Rodríguez-Negrete et al. (2019)
Watermelon chlorotic stunt virus	WmCSV	*Begomovirus citrulli*	*Begomovirus*	Watermelon (*Citrullus lanatus*)	Hernández-Pérez et al. (2025)
Watermelon mosaic virus	WMV	*Potyvirus citrulli*	*Potyvirus*	Watermelon (*Citrullus lanatus*)	Lopez-Roblero et al. (2023)

In maize and its wild relative teosinte/teocintle (*Zea* spp.), the application of HTS enabled the discovery of four novel viruses in North America, including the first *Mastrevirus* identified in this region (Lappe et al., 2022). Additionally, this study revealed the presence of new viral species of *Betaflexviridae, Tombusviridae* and *Geminiviridae* families. In the case of beans, an essential crop for food security in Mexico, HTS has detected the presence of multiple viruses, including *Phaseolus vulgaris* alphaendornavirus 1 (PvAEV1, *Alphaendornavirus phaseoli*) and common bean severe mosaic virus (CBSMV, *Begomovirus vulgaris*; Chiquito-Almanza et al., 2021). The detection of these viruses in domesticated and wild plants has allowed a better understanding of the evolution of infectious agents in these plants and its potential impact on agricultural productivity.

A successful case in which HTS allowed the identification of viruses in Mexico was in agave (De la Torre-Almaraz et al., 2023). The detection of tuberose mild mosaic virus (TMMV, *Potyvirus polianthis*) in *A. attenuata* and *A. amica* using this technology represented a relevant finding since these plants had not been previously reported as hosts of this potyvirus. This discovery emphasizes the need to continue monitoring the viromes of perennial crops to prevent the spread of emerging pathogens. Agave is used to produce tequila, one of the main products exported by Mexico.

In the case of grapevine, HTS has allowed the characterization of many viruses that affect this plant in Mexico (Diaz-Lara et al., 2023). Viruses such as grapevine leafroll-associated virus 4 (GLRaV-4, *Ampelovirus tetravitis*), grapevine Pinot gris virus (GPGV, *Trichovirus pinovitis*) and grapevine Syrah virus 1 (GSyV-1, *Marafivirus syrahense*) have been identified. These findings contribute valuable information that could support improvements in sanitary certification programs, although their implementation in official management practices has yet to be documented in Mexico.

Another relevant case is that of the prickly pear (*Opuntia* spp.), a crop of great nutritional and ecological importance in Mexico. Through HTS, Ortega-Acosta et al. (2024a,b) managed to identify the viroids Opuntia cactus viroid 1 (OCVd-1), Opuntia viroid 2 (OVd-2), as well as a novel virus in this plant. This study represents a fundamental starting point for monitoring and preventing viral diseases in the prickly pear, ensuring its sustainable production and reducing possible economic losses.

Tomato is a crop of significant economic importance in the country and have also been subject of extensive analysis. Emerging variants of pepino mosaic virus (PepMV, *Potexvirus pepini*) have been identified (Miranda-Campaña et al., 2024), and the genetic diversity of tomato brown rugose fruit virus (ToBRFV, *Tobamovirus fructirugosum*), a virus that has caused severe losses in tomato production in Mexico and other countries, has been investigated (Cambrón-Crisantos et al., 2018). Understanding the genetic variability of these viruses is useful toward improved detection assay design.

In bell pepper (*Capsicum annuum*), mixed infections caused by Impatiens necrotic spot virus (INSV, *Orthotospovirus impatiensnecromaculae*) and ToBRFV has been determined by HTS (De Mora-Ugalde et al., 2024). Similarly, mixed infections induced by cucurbit yellow stunting disorder virus (CYSDV, *Crinivirus cucurbitae*) and watermelon chlorotic stunt virus (WmCSV, *Begomovirus citrulli*) were detected in watermelon (*Citrullus lanatus*) in Jalisco, Mexico (Hernández-Pérez et al., 2025). These co-infections underline the complexities of viral epidemiology in intensive production systems and emphasize the necessity for management strategies that incorporate advanced molecular diagnosis, including multiplex assays.

In papaya, a crop of significant economic importance in southern Mexico, HTS and network analysis revealed a highly diverse virome composed of both known and novel viruses (Alcalá-Briseño et al., 2020). The study identified 61 viral agents, across papaya plants, weeds, and insect vectors in two agroecological regions of Chiapas. The presence of viruses in asymptomatic papayas and surrounding weeds emphasized the complexity of virus-host interactions. Likewise, Rodríguez-Negrete et al. (2019) identified multiple species of begomoviruses in wild plants in the Northern Pacific of Mexico, which shows that these weeds can act as natural reservoirs of pathogens that can potentially migrate to commercial crops.

In addition to food crops, HTS has been used to identify viruses in species with pharmacological potential. An example is the case of *Galphimia* spp., where the sequencing technology detected the Galphimia cryptic virus (GCV) for the first time (Iglesias et al., 2024). This work demonstrates that HTS is not only useful in agricultural plants, but also in species of medicinal interest for Mexico.

Meta-analysis of water using HTS has shown its role as a source of virus dissemination in agricultural systems. A study by Lopez-Roblero et al. (2023) detected a high diversity of viruses in tropical water bodies used for agricultural irrigation. Among the viruses detected are lettuce chlorosis virus (LCV, *Orthotospovirus impatiensnecromaculae*), papaya ringspot virus (PRSV, *Potyvirus papayanuli*), and tomato yellow leaf curl virus (TYLCV, *Begomovirus coheni*), all of them pathogens of great agricultural importance (Alabi et al., 2017). The presence of these viruses in water sources reinforces the notion that aquatic systems can function as routes of virus dispersion, representing a latent risk for the spread of diseases in crops.

## 3 Challenges in diagnosing plant viruses by high-throughput sequencing in Mexico

The use of HTS has facilitated the detection and identification of viruses in plants, enabling researchers to characterize new viral species and analyze complex interactions. However, its implementation faces various technical, economic, and methodological challenges that limit its large-scale application in developing countries like Mexico.

One main obstacle is limited infrastructure access and adequate equipment for generating and analyzing HTS data. Unlike other more traditional diagnostic approaches, HTS requires advanced technology, such as high-throughput sequencers (e.g., Illumina, Oxford Nanopore or PacBio) and high-performance computing platforms for data processing, like computer clusters (servers; Mehetre et al., 2021; Satam et al., 2023). This represents a barrier for many laboratories in Mexico, especially in institutions with limited funding or regions with little technological infrastructure.

Bioinformatics analysis is another of the main challenges in HTS research since the massive volume of data generated requires advanced algorithms built upon programming (i.e., bioinformatic pipeline) and personnel trained in bioinformatics and computational biology (Wu et al., 2015; Valenzuela et al., 2022). Researchers often face difficulties in the selection and application of appropriate bioinformatics tools for the assembly of viral genomes, the removal of contaminating data, and the interpretation of newly emerged sequences (Rodríguez-Negrete et al., 2019; Maina et al., 2024). Furthermore, the lack of updated and/or validated data in public databases like GenBank for sequence comparison complicates accurately identifying emerging or poorly characterized viruses and viroids (Rivarez et al., 2023; Ning et al., 2024).

Another major obstacle is the biological interpretation of the results obtained through HTS. Although this technology allows the detection of a wide diversity of viruses and viroids in environmental or plant samples, the presence of viral sequences does not always imply active infection or relevant phytopathological impact (Elena et al., 2014; Picard et al., 2017). This can generate confusion in decision-making for phytosanitary management and underlines the need to complement HTS with additional biological and experimental studies, for example, virus transmission (Diaz-Lara et al., 2023; Ortega-Acosta et al., 2024a,b).

Finally, the high costs associated with HTS remain a major limitation to its widespread adoption. Although sequencing costs have decreased over the past decade, sample processing and library preparation, data storage and analysis, and the validation of results using conventional-traditional methods can represent significant expenses for researchers (Nizamani et al., 2023; Kanapiya et al., 2024). This highlights the need for sustainable funding strategies and international collaborations to strengthen virological research in Mexico.

## 4 Future perspectives on the use of HTS for virus diagnosis in Mexico

The use of HTS for virus and viroid detection in plants in Mexico represents an innovative tool with great potential to improve plant health. This technology can simultaneously detect a wide range of plant viruses, including those present in low concentrations or without obvious symptoms, makes it a key strategy for epidemiological monitoring and the management of viral diseases in crops (Al Rwahnih et al., 2015; Rott et al., 2017; Maree et al., 2018). Currently, virus diagnosis in Mexico is relying primarily on traditional methods such as PCR and ELISA. While these methods are specific and accessible, they require prior knowledge of the virus to be detected (Roossinck et al., 2015). In contrast, HTS allows the identification of known and unknown viruses without the need for previously published information, making it an indispensable technique for monitoring emerging viruses and detecting new viral variants (Villamor et al., 2019). Consequently, in Mexico, where plant viral diseases significantly threaten agricultural productivity, the implementation of HTS has the potential to transform virus diagnosis and management strategies.

Looking to the future, Mexico will face multiple issues related to plant virology. The increasing incidence of emerging viruses, particularly tospoviruses and begomoviruses, transmitted by thrips and whiteflies, respectively; these types of pathogens have increased their impact on the country’s horticultural production and severely affect staple and economically important crops such as tomatoes, peppers, and beans (Rodríguez-Negrete et al., 2019). Traditional diagnostic methods used in Mexico, including serological assays and PCR-based techniques, often fail to detect mixed infections or new viral variants, limiting the effectiveness of disease management strategies. The application of HTS will allow for a more comprehensive analysis of viral populations, allowing the detection of complex viral co-infections that influence disease epidemiology.

While sample processing, library preparation, data generation and storage, and result validation may represent significant expenses for Mexican researchers, several alternatives can help reduce costs. These include the use of low-cost library prep kits, shared access to sequencing services through academic consortia or private providers, cloud services for data storage, and free web-based platforms for bioinformatic analysis (e.g., VirFind and Viroscope). Lastly, targeted PCR validation of HTS-detected sequences offers a cost-effective method to confirm key findings.

To fully integrate HTS into plant health programs in Mexico, regulatory agencies such as SENASICA and State Plant Health Committees must adopt standardized-validated protocols for sample preparation, sequencing and later data interpretation. Currently, SENASICA oversees phytosanitary measures to control the spread of plant pathogens, and a greater reliance on HTS would improve early detection and rapid response capabilities, especially at points of entry into the country. Furthermore, collaborations between research institutions, public and private, and regulatory agencies could facilitate the development of national reference databases for plant viruses and viroids, improving diagnostic accuracy and epidemiological monitoring.

## 5 Conclusion

The application of HTS in plant virology in Mexico has driven significant advances in the detection and management of viruses and viroids affecting key agricultural crops. This approach enables the identification of mixed infections, new viral species, and a deeper understanding of viral diversity in agroecosystems. Its impact on plant health is unquestionable, offering more sensitive and efficient diagnoses than traditional techniques.

The increased use of HTS in Mexican agricultural systems is improving the characterization of emerging viruses and viroids, understanding their symptoms, and faster outbreak responses. Collaboration between institutions and regulatory bodies remains essential to harmonize diagnostic protocols and ensure reliable results. Incorporating HTS as a routine tool in regulatory bodies such as SENASICA would strengthen national viral disease monitoring systems in strategic crops. Thus, the integration of HTS into national plant health programs, such as certification schemes, would improve epidemiological responses, particularly through collaboration between research centers, government agencies, and industry.

Finally, while the adoption of HTS in Mexico offers multiple benefits, it faces barriers such as cost, limited equipment accessibility, and insufficient frameworks for data sharing. Overcoming these obstacles will require investment in infrastructure, personnel training, and policy development to incorporate genomics into standard diagnostics. Importantly, these challenges are not unique to Mexico, similar conditions likely exist in other developing countries, where scarce resources and knowledge can hinder HTS implementation, despite comparable plant health surveillance needs. However, exploring HTS applications opens promising avenues for identifying resistance genes, establishing resilient crops, and reducing the impact of viral diseases, thus boosting sustainable agriculture. While the task is complex, we are moving in the right direction.

## Data Availability

The original contributions presented in the study are included in the article/supplementary material, further inquiries can be directed to the corresponding author.

## Footnotes

^1^ https://cicese-at.cicese.mx/archivos/2017/La_Virologia_en_Mexico_Situacion_Actual_4.pdf
